# Pain Reduction in Cervical Dystonia Following Treatment with IncobotulinumtoxinA: A Pooled Analysis

**DOI:** 10.3390/toxins15050333

**Published:** 2023-05-12

**Authors:** Alberto Albanese, Jörg Wissel, Wolfgang H. Jost, Anna Castagna, Michael Althaus, Georg Comes, Astrid Scheschonka, Matteo Vacchelli, Hyder A. Jinnah

**Affiliations:** 1 Department of Neurology, IRCCS Humanitas Research Hospital, 20089 Rozzano, MI, Italy; 2 Department of Neurorehabilitation and Physical Therapy, Vivantes Hospital Spandau, 13585 Berlin, Germany; joerg@schwarz-wissel.de; 3 Parkinson-Klinik Ortenau, 77709 Wolfach, Germany; w.jost@parkinson-klinik.de; 4 IRCCS Fondazione Don Carlo Gnocchi, 20148 Milano, MI, Italy; acastagna@dongnocchi.it; 5 Merz Therapeutics GmbH, 60318 Frankfurt am Main, Germany; michael.althaus@merz.de (M.A.); georg.comes@merz.de (G.C.); astrid.scheschonka@merz.de (A.S.); matteo.vacchelli@merz.de (M.V.); 6 Department of Neurology, Emory University School of Medicine, Atlanta, GA 30322, USA; hjinnah@emory.edu

**Keywords:** botulinum toxin type A, cervical dystonia, incobotulinumtoxinA, pain, pooled analysis

## Abstract

This analysis pooled pain severity data from four phase 3 and 4 studies of incobotulinumtoxinA (incoBoNT-A) for the treatment of cervical dystonia (CD) in adults. CD-related pain severity was assessed at baseline, each injection visit, and 4 weeks after each injection of incoBoNT-A using the Toronto Western Spasmodic Torticollis Rating Scale pain severity subscale or a pain visual analog scale. Both were analyzed using a score range of 0–10 and pain was categorized as mild, moderate, or severe. Data for 678 patients with pain at baseline were assessed and sensitivity analyses evaluated pain responses in the subgroup not taking concomitant pain medication (*n* = 384 at baseline). At Week 4 after the first injection, there was a mean change of −1.25 (standard deviation 2.04) points from baseline pain severity (*p* < 0.0001), with 48.1% showing ≥ 30% pain reduction from baseline, 34.4% showing ≥50% pain reduction from baseline, and 10.3% becoming pain free. Pain responses were sustained over five injection cycles with a trend to incremental improvements with each successive cycle. Pain responses in the subgroup not taking concomitant pain medication demonstrated the lack of confounding effects of pain medications. These results confirmed the pain relief benefits of long-term treatment with incoBoNT-A.

## 1. Introduction

Cervical dystonia (CD), a chronic neurological disorder characterized by involuntary contractions of cervical muscles, causes abnormal resting positions and movements of the head, neck, and/or shoulders. The term isolated CD (iCD) is used when other movement disorders, except tremors, are not involved [1]. CD is the most common form of adult-onset focal dystonia, with a pooled prevalence estimate of 9.95 cases per 100,000 in a recent meta-analysis of epidemiological studies [2].

Patients with CD experience non-motor symptoms such as pain, functional impairment, and anxiety/depression, which negatively affect their daily activities and quality of life (QoL) [3,4]. Pain is the most common non-motor symptom of CD, affecting 55–90% of patients with CD and is rated as moderate or severe by 71% of patients [5,6,7,8,9]. There is increasing evidence that pain in CD is not only caused by muscle overactivity, especially spasm of the larger neck muscles, but that other mechanisms such as abnormal transmission and processing of nociceptive stimuli, alteration of pain inhibition via the descending pathway, and structural changes may be involved [9]. Pain is often the main reason patients with CD seek treatment [7,8,9] and is a major contributor to disability [10,11] and impaired QoL [5,6]. There are no specific criteria for pain classification in CD, and few scales specifically assess CD-related pain and its impact on disability; thus, the clinical assessment and management of CD-related pain could be improved [12].

Botulinum toxin type A (BoNT-A) is recommended as the first-line treatment for CD [13,14,15,16,17]. Several BoNT-A formulations are available for the treatment of CD, of which the three approved in the USA are abobotulinumtoxinA (aboBoNT-A), onabotulinumtoxinA (onaBoNT-A), and incobotulinumtoxinA (incoBoNT-A) [17]. BoNT-A is administered by intramuscular injection into the overactive muscles, and pain that is improved by BoNT-A injections is one of the supportive criteria for a diagnosis of iCD [1]. A single treatment session effectively reduces CD symptoms, but the symptoms re-emerge over time such that repeated injections are needed at approximately 3-month intervals for sustained benefits [18].

The efficacy and tolerability of incoBoNT-A—a highly purified preparation that is free from accessory proteins—has been demonstrated in patients with CD in two pivotal phase 3 studies [19,20,21]. Subgroup analyses showed similar efficacy in patients naïve to BoNT-A and those previously treated with onaBoNT-A [22].

The efficacy of BoNT-A in relieving pain in patients with CD has been confirmed in analyses of controlled clinical trial data [9,23] and in large-scale observational studies/registries of CD patients (CD-PROBE, ANCHOR-CD) [4,24,25,26]. Nevertheless, the pain response profile to BoNT-A in CD is not yet fully understood. More information is needed on numerous factors, including the severity of CD-related pain, the short- and long-term effects of BoNT-A on pain reduction/relief, the time-course of pain reduction, and the effects of CD-related pain and BoNT-A treatment on patient-reported outcomes such as disability and QoL.

The aim of this analysis was to investigate the impact of incoBoNT-A on pain related to CD by pooling data from four incoBoNT-A studies in the treatment of CD in adults [19,20,27,28].

## 2. Results

Across the four studies, 1054 patients were treated with incoBoNT-A at least once. Patients receiving placebo or onaBoNT-A in the first injection cycle (*n* = 306) received incoBoNT-A in the second and subsequent injection cycles, if performed. Data from these incoBoNT-A cycles were included in the analyses such that the first injection cycle with incoBoNT-A was designated injection cycle 1.

Of the 1054 patients treated with incoBoNT-A, 329 (31.2%) had no pain assessment at baseline, 47 (4.5%) had no pain at baseline, and the remaining 678 (64.3%) patients reported having pain at baseline and formed the basis of this pooled analysis. Of the 678 patients with pain at baseline, 247 (36.4%) had mild pain, 291 (42.9%) had moderate pain, and 140 (20.6%) had severe pain (Table 1). The patients with pain at baseline had a mean age of 53.6 years, 68.3% were female, 21.4% had severe disease based on a Toronto Western Spasmodic Torticollis Rating Scale (TWSTRS) severity score of 22–35, and 18.1% were BoNT-A naïve (Table 1).

The patient numbers at each injection visit (IV) and control visit (CV; 4 weeks post-injection) for 5 injection cycles for the total population and by individual study are shown in Figure S1. As the data for CV6 and subsequent injection cycles were provided by only one study, we did not include the results for injection cycle 6 in our presentation of the pooled data. The number of patients with pain assessment in each injection cycle is given in Table S1.

### 2.1. Change in Pain Severity from Baseline to Week 4

**Figure 1:**
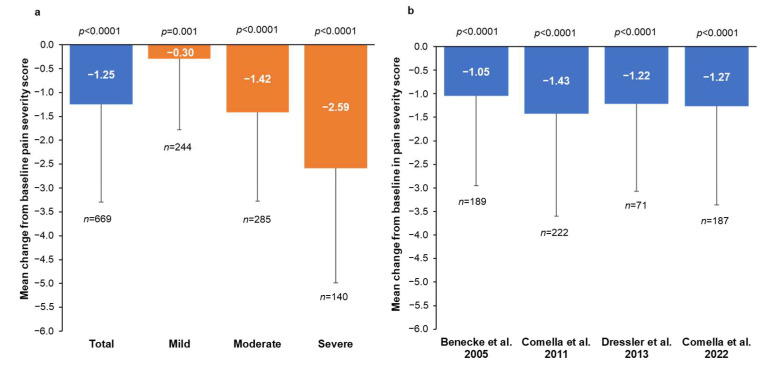

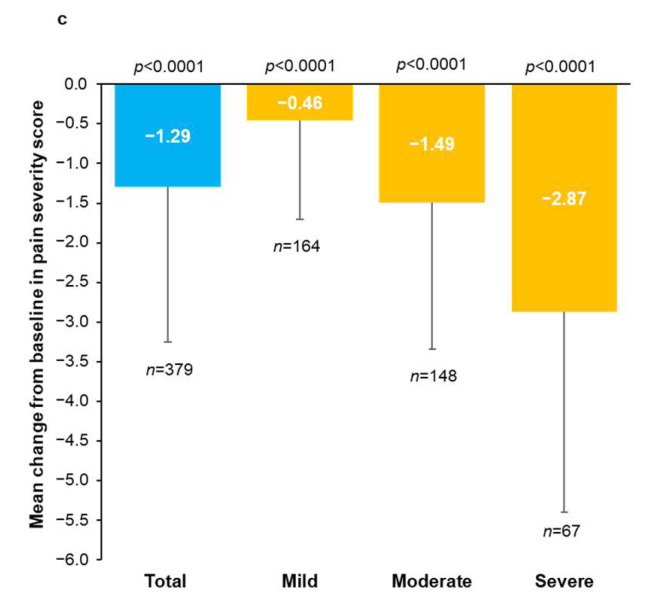
Mean change from baseline in pain severity at control visit 1 (CV1) 4 weeks after the first injection of incobotulinumtoxinA for: (**a**) the total population with pain assessment at CV1 (*n* = 669) and by baseline pain severity (mild, moderate, severe); (**b**) by individual study [19,20,27,28]; and (**c**) for patients with pain assessment at CV1 not taking concomitant pain medication (*n* = 379). Pain severity score ranges from 0 to 10. Bars represent standard deviation. *p*-values are from one-sample *t*-tests.

At baseline, the mean (standard deviation [SD]) pain severity score was 4.26 (2.32) for the total population (*n* = 678) and 1.73 (1.06), 4.91 (0.80), and 7.40 (0.83) for the mild, moderate, and severe pain groups, respectively (Table 1). Patients treated with incoBoNT-A showed a significant reduction in the mean pain severity score at CV1 (Figure 1). There was a mean (SD) change of −1.25 (2.04) points from baseline pain severity (*p* < 0.0001) in the total population with pain data at CV1 (*n* = 669) (Figure 1a) and this change was consistent across all four individual studies (Figure 1b).

**Figure 2:**
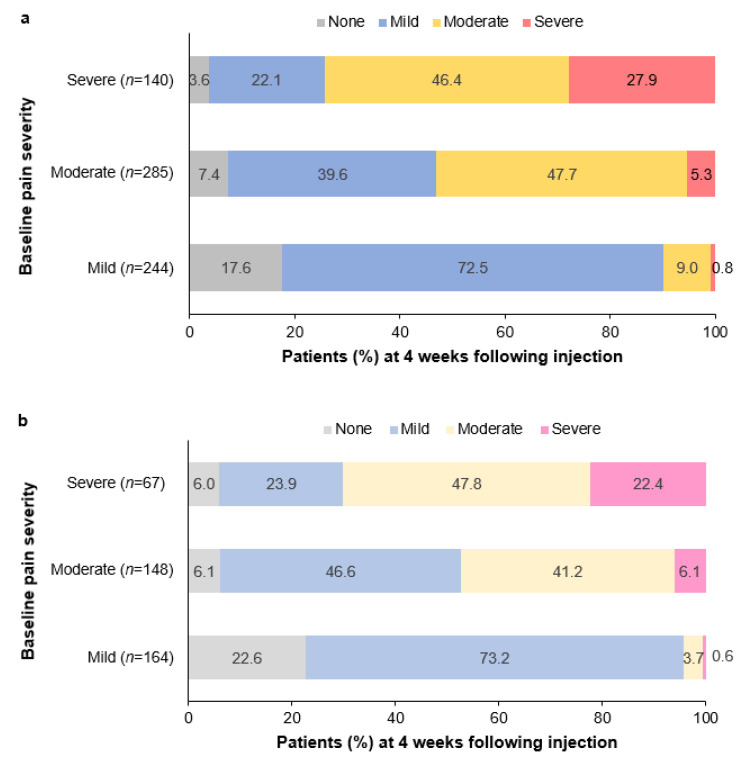
Change in pain severity at control visit 1 (CV1) 4 weeks following a single injection of incobotulinumtoxinA by baseline pain severity in: (**a**) total study population (*n* = 669) and (**b**) subgroup of patients not taking concomitant pain medication (*n* = 379). Each stacked bar shows the percentage of patients in each pain category at CV1 based on the numbers of patients in the baseline pain severity category. Total percentages may be 100 ± 0.1% due to rounding.

Change in pain severity at Week 4 (CV1) following a single injection of incoBoNT-A by baseline pain severity category is presented in Figure 2a. The figure shows a shift to a lower level of pain severity at Week 4 for a good proportion of the patients in each of the baseline pain categories. Of the 244 patients with mild pain at baseline, 43 (17.6%) had no pain at CV1. Of the 285 patients with moderate pain at baseline, 21 (7.4%) had no pain and 113 (39.6%) had mild pain at CV1. Of the 140 patients with severe pain at baseline, 5 (3.6%) had no pain, 31 (22.1%) had mild pain, and 65 (46.4%) had moderate pain at CV1. Notably, 10.3% of all patients with pain at baseline (mild, moderate, or severe) reported no pain at Week 4. Additionally, the proportion of patients with mild pain increased from 36.5% at baseline to 48.0% at Week 4, while the proportion with moderate pain decreased from 42.6% at baseline to 33.3% at Week 4, and the proportion with severe pain decreased from 20.9% at baseline to 8.4% at Week 4 (Figure S2a).

### 2.2. Pain Severity, Response Rates, and Complete Pain Relief Following Multiple Injection Cycles

Changes in pain severity by category of pain severity were maintained over multiple injection cycles (Figure S3). Of the 669 patients with pain data at CV1, 322 (48.1%) patients had ≥30% pain reduction from baseline and 230 (34.4%) patients had ≥50% pain reduction from baseline; these patients were classified as responders.

**Figure 3:**
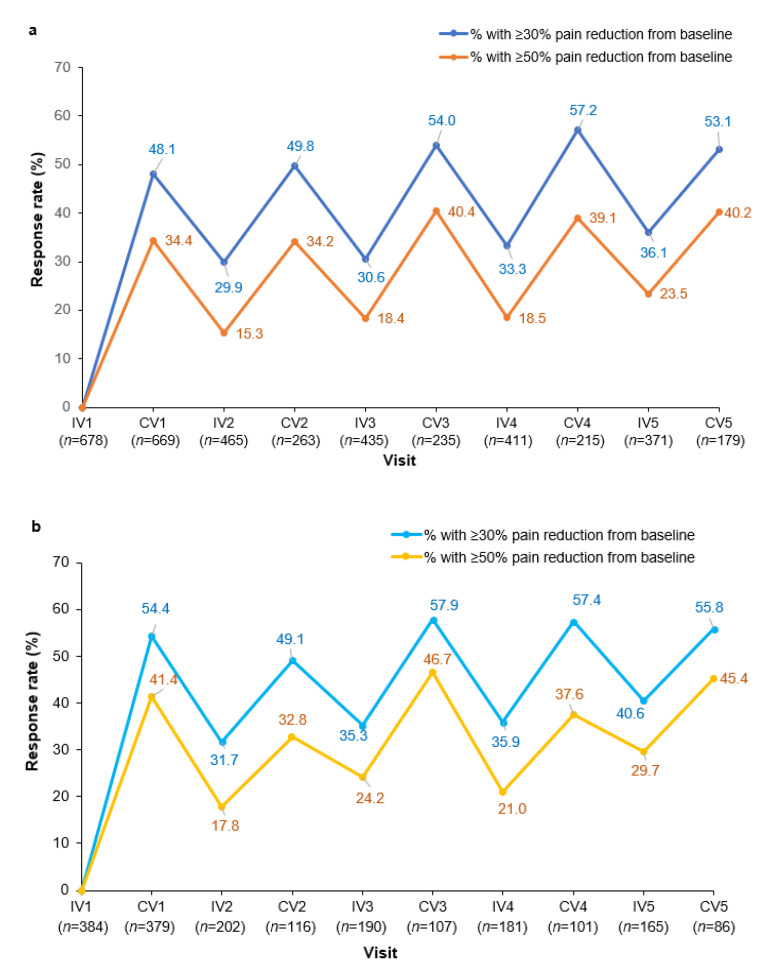
Response rates defined as ≥30% or ≥50% pain reduction from baseline (IV1) over repeated injection cycles of incobotulinumtoxinA for: (**a**) total study population and (**b**) subgroup without any concomitant pain medication. The *n* value given for each visit is the number of patients with data available for that visit. IV, injection visit; CV, control visit.

Pain response rates (≥30% or ≥50% reduction in pain score from baseline) were sustained following multiple injections of incoBoNT-A (Figure 3a). The results also showed that pain had not returned to the baseline level by the time of their next injection, demonstrating a cumulative effect throughout the cycles. In total, 29.9% at IV2 to 36.1% at IV5 were classified as responders (≥30% pain reduction from baseline) and 15.3% at IV2 to 23.5% at IV5 were classified as responders (≥50% pain reduction from baseline) (Figure 3a).

**Figure 4:**
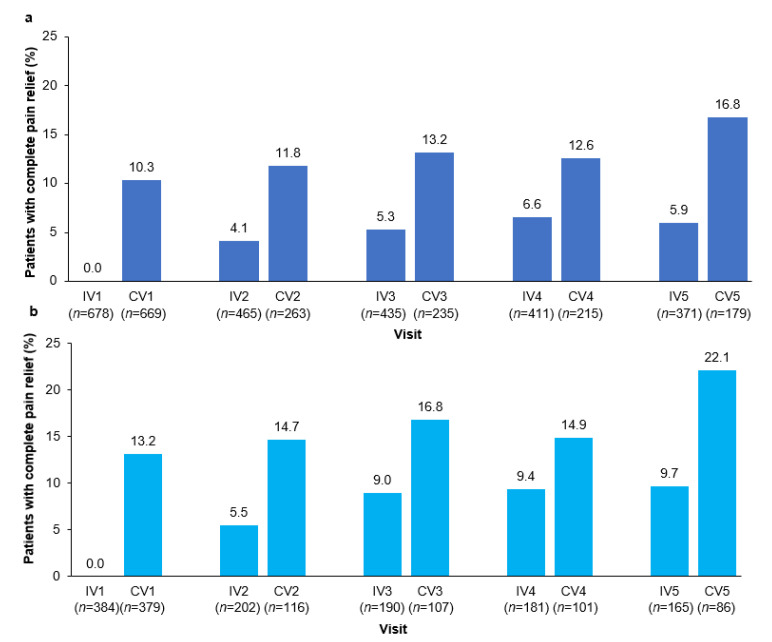
Proportion of patients achieving complete pain relief with incobotulinumtoxinA over time in: (**a**) total population and (**b**) subgroup not taking concomitant pain medication. The *n* value given below each visit is the number of patients with data available for that visit. CV, control visit; IV, injection visit.

The proportion of patients with complete pain relief at the control visit increased slightly over the first 5 injection cycles from 10.3% at CV1 to 16.8% CV5 (Figure 4a). Notably, a small proportion of patients were pain free at the time of the second and subsequent injection visits.

### 2.3. Pain Severity Responses in Patients Not Taking Concomitant Pain Medication

Of the 678 patients with pain at baseline, 435 (64.2%) were not taking concomitant pain medication at IV1. The proportions of patients taking any pain medication were similar at IV1 and CV1 (Table S2), and 9% of patients received benzodiazepines or muscle relaxants at IV1 and CV1. There was no trend towards increased use of analgesic medication during the study. The pain severity responses in the subgroups of incoBoNT-A-treated patients with pain at baseline and patients not taking concomitant pain medication were consistent with the findings presented above for patients with pain at baseline regardless of concomitant pain medication. These results indicated that concomitant pain medication did not influence the pain response rates. At IV1, the mean (SD) pain severity score was 3.83 (2.41), with 43.2% reporting mild pain, 39.3% reporting moderate pain, and 17.5% reporting severe pain in the subgroup not taking concomitant pain medication (*n* = 384). The mean (SD) change in pain severity score from baseline at CV1 following a single injection of incoBoNT-A for the subgroup without concomitant pain medication and pain data at CV1 (*n* = 379) was −1.29 (1.96; *p* < 0.0001) (Figure 1c). The change in pain severity at CV1 by baseline pain severity category (Figure 2b) showed a shift towards less severe pain. Of the 379 patients with pain at IV1 (mild, moderate, or severe) and pain data at CV1, 50 (13.2%) reported no pain at CV1 (Figure S2b). Additionally, the number of patients (%) with mild pain increased from 164 (43.3%) at IV1 to 205 (54.1%) at CV1, while there were decreases in the numbers (%) of patients with moderate pain from 148 (39.1%) to 99 (26.1%) and with severe pain from 67 (17.7%) to 25 (6.6%) (Figure S2b).

The response rates at CV1 among patients not taking concomitant pain medication were 54.4% (≥30% pain reduction from baseline) and 41.4% (≥50% pain reduction from baseline), and the response rates remained high over subsequent injection cycles (Figure 3b).

The proportion of patients not taking concomitant pain medication with complete pain relief increased from 13.2% at CV1 to 22.1% at CV5 (Figure 4b), showing a cumulative effect over time. The proportion of patients who remained pain-free at the time of their next injection of incoBoNT-A ranged from 5.5% to 9.7%.

## 3. Discussion

In this pooled analysis, we investigated the clinical course of pain severity in a large cohort of patients with CD from a wide geographic area (Europe and USA) treated with incoBoNT-A. We observed significant pain reduction during the first injection cycle, which was sustained throughout several treatment cycles. These reductions in pain severity were also seen in patients who had already been successfully treated with BoNTs before study enrolment (82% of cases in this analysis). Pain reduction/relief was also consistently observed in the subgroup of patients not taking concomitant pain medication, confirming the benefits of long-term treatment with incoBoNT-A. Effective pain control in CD is important to prevent the development of chronic pain [29].

As expected, a high proportion of patients had pain at baseline (64%), which was moderate or severe in 64% of these patients. Our total population of incoBoNT-A-treated patients with pain at baseline comprised mostly middle-aged women (68% women, mean age 53.6 years), consistent with the prevalence data that women are approximately two times more likely than men to have CD and the mean age of CD onset is 42 years [30].

Our results showed a reduction in pain severity after treatment with incoBoNT-A. At Week 4 after the first injection, we observed a mean reduction of 1.25 points in the pain severity score from baseline (score range 0–10), and a large proportion of patients met the response criteria: 48.1% with ≥30% pain reduction from baseline level and 34.4% with ≥50% pain reduction from baseline level. The pain reduction shown by this analysis can be considered clinically relevant, as it is consistent with the following criteria. The CD-PROBE study suggested that a 2-point change in the TWSTRS pain score (score range 0–20) could be considered a minimum clinically important change [31], and the Initiative on Methods, Measurement, and Pain Assessment in Clinical Trials (IMMPACT) recommendations on chronic pain [32] state that reductions in pain intensity of ≥30% and ≥50% appear to reflect at least moderate or substantial clinically important improvements.

The observed reduction in pain severity was sustained over repeated injections of incoBoNT-A and there was a trend over the five treatment cycles towards incremental pain reduction/relief. These results showed that even if pain was still present at the control visit (4 weeks post-injection), it was less severe than at the injection visit of that treatment cycle. Moreover, the proportion of patients with mild pain increased, while the proportion of patients with moderate or severe pain decreased relative to that at the injection visit. Importantly, we found that 10.3% of patients with pain at baseline were pain-free at Week 4 after the first incoBoNT-A injection, and the proportion of patients who experienced complete pain relief increased over each subsequent injection cycle to 16.8% at the 5th injection cycle. As only 18% of the patients in our analyses were BoNT naïve, previous treatment with other BoNTs may have attenuated the analgesic effect of incoBoNT-A.

Previous studies of BoNT-A treatment in CD have shown persistent reductions in pain during long-term treatment with aboBoNT-A [33,34], onaBoNT-A [5,35], and incoBoNT-A [21,27,28] when pain was scored using the TWSTRS and/or a pain numeric rating scale. A ≥30% improvement from baseline in pain score was also noted in a large cohort of patients with moderate-to-severe pain treated with onaBoNT-A in the CD-PROBE observational study [24] and in a meta-analysis of observational studies of aboBoNT-A in patients with CD [26], supporting the use of ≥30% reduction in pain as a clinically relevant measure.

Pain severity was assessed using established and validated pain scales (TWSTRS severity subscale and pain visual analog scale [VAS]) that are widely used in clinical trials and have been shown to correlate well with each other [5]. The TWSTRS covers a range of CD features, including disease severity, functional ability, and pain [36]. IncoBoNT-A has been shown to effectively control CD symptoms after each injection, as reflected by improvements in the TWSTRS total score and TWSTRS subscale scores for motor severity, disability, and pain [19,20,21,27,28]. However, the TWSTRS does have several drawbacks, including its complexity for use in clinical practice and it does not consider the location of pain [36], which may lead to an underestimation of pain severity.

The TWSTRS pain subscale not only measures pain severity but also the duration of pain and the contribution of pain to disability, but these were inconsistently assessed across the studies included in the pooled analysis. Therefore, these factors were not analyzed. We also did not examine changes in the TWSTRS disability score in this pooled analysis. However, it is well known that pain contributes to disability in CD [11] and that BoNT treatment, including incoBoNT-A, reduces both pain and disability [20,21,27,28,37]. A recent case-control study found that patients with CD-related pain had significantly higher TWSTRS severity and disability scores than those without pain and that pain reduction following BoNT injection lasted longer than muscle relaxation in 85.3% of patients with pain, although muscle relaxation preceded pain improvement for 53% of these patients [8]. As we examined changes in pain severity using TWSTRS severity subscale or pain VAS scores, comparing our data with those of other studies is difficult. However, despite different patient populations and methods of assessing pain, clinical studies with other BoNT-A formulations in CD have reported changes in pain severity that are consistent with the mean change of −1.25 on a scale of 0–10 reported for incoBoNT-A in our analysis (Figure 1a). For example, in a pooled analysis of data for aboBoNT-A in CD, an adjusted mean change of −3.2 ± 0.2 points at Week 4 was reported for aboBoNT-A using the TWSTRS pain score (scale range 0–20) [9], with a separate study reporting a mean change of −3.7 points from baseline at Week 4 for aboBoNT-A using the TWSTRS pain subscale score (scale range 0–20) [33].

Many CD patients report having re-emergent motor symptoms before the next BoNT-A injection. In the Carenity survey, 88% of patients reported symptom re-emergence at approximately 10.5 weeks from injection [38]. The benefits of BoNT-A start to wear off after about 8 weeks, creating a cyclical response known as the “yo-yo” effect [39] that may contribute to patient dissatisfaction, reduced QoL, and discontinuation of therapy [40]. The “yo-yo” effect varies between individuals and by the BoNT-A preparation used [39]. A novel finding from our analyses was that CD-associated pain demonstrated this “yo-yo” effect across BoNT-A injection cycles. Re-emergent pain before the next injection is likely to have an impact on patients. In the Carenity patient survey, pain was one of the first symptoms to re-emerge as the effects of BoNT-A wore off, and this waning effect had an impact on the patient’s ability to work, their daily activities, and QoL [38]. Our results showed that although around 5% of patients remained pain free at the time of their next incoBoNT-A injection (Figure 4a), pain levels increased between CV and the next IV, as reflected in the lower proportion of patients with ≥30% or ≥50% pain reduction at the next IV (Figure 3a). Indeed, the increasing proportion of patients classified as responders at successive injection visits implied progressive improvements in pain with repeated incoBoNT-A treatments. It would be interesting to further explore these findings of a cyclical pattern of pain reduction and recurrence between incoBoNT-A injections as CD therapy aims to reduce pain or keep patients pain-free.

Pain is also an important determinant of QoL in patients with CD and can be assessed using various scales. The CDQ-24, a 24-item disease-specific health-related QoL measure, includes a pain subscale that has demonstrated sensitivity to change following BoNT treatment and good correlation with other pain rating scales [41,42]. The CDQ-24 pain scale also measures frequency of pain, which is not measured by the TWSTRS, and is useful for assessing the impact of pain on QoL. Further clinical studies on pain reduction in CD with BoNT therapy should include both scales.

Pain is strongly associated with emotional well-being and depression [43] and is a relevant contributor to the severe negative impact of CD on health and disability as it limits patients’ activities and participation, as measured using the International Classification of Functioning, Disability, and Health [44]. Higher pain scores (pain VAS and TWSTRS pain score) have been associated with worse patient satisfaction with BoNT-A treatment [45], but patient satisfaction is a different measure from pain rating and we did not assess patient satisfaction in the current pooled analysis. Although we focused on pain severity, we can speculate that the reduction in pain severity seen with incoBoNT-A injections is likely to be associated with greater patient satisfaction with treatment, less disability, and improved QoL.

It has been reported that approximately two-thirds of patients with CD use oral/systemic analgesics to manage their CD-related pain [46,47]. In our pooled analysis, only 36% of patients with pain at baseline reported using concomitant pain medication at the first injection visit. This may be because most participants (82%) had already been successfully treated with BoNTs before study enrolment, which would contribute to an underestimation of the pain-relieving effect of incoBoNT-A.

Our results in the subgroup of CD patients with pain not taking pain medication (about two-thirds of the total population) confirmed that the pain reduction/relief we observed was a result of incoBoNT-A treatment and not due to analgesic medication.

The limitations of our analysis include potential bias due to different patient numbers and varied numbers of injection cycles in the different studies used in the pooled analysis. The effect of incoBoNT-A on pain may have been underestimated because most (82%) patients had been previously treated with other BoNTs. Additionally, pain was assessed as a secondary outcome measure in all four studies, and pain assessment at the control visit (4 weeks after injection) may not have been the peak time of pain relief, potentially underestimating the effect of incoBoNT-A on reducing pain. The scales used to assess pain in the studies included in the pooled analysis did not give a full profile of pain, so we focused on pain severity. One study in the pooled analysis used a pain VAS and, even though the pain VAS scores were transformed to a 0–10 scale, the pain VAS may not have provided an identical measure of pain severity as the TWSTRS severity subscale. Nevertheless, a pain VAS is recommended by IMMPACT for use in pain studies to measure pain intensity and the effectiveness of treatments [32]. Patients with no pain at baseline were excluded from our pooled analysis, but it is possible that some of these patients developed pain during the study.

## 4. Conclusions

This pooled analysis showed that patients with CD-related pain experienced clinically relevant and sustained reductions in pain during repeated incoBoNT-A injections, with a trend toward greater pain relief over successive treatment cycles. The level of pain after incoBoNT-A injection was reduced in severity for many patients, leading to increases in the proportions of patients with mild or no pain. After repeated injections, 16.8% of patients became pain-free at 4 weeks post-injection. The pain reduction/relieving effects of incoBoNT-A were also observed in the large subgroup of patients not taking concomitant pain medication. Our results provide further evidence to support the long-term use of incoBoNT-A for pain relief in patients with CD. Active questioning about CD-related pain in clinical practice may be important for the timing of incoBoNT-A re-injection and to improve pain management, thereby avoiding cycles of pain relief and recurrence.

## 5. Materials and Methods

### 5.1. Studies Included in the Pooled Analysis

Analysis was based on the pooled results from four prospective, multicenter, phase 3 and 4 studies conducted for incoBoNT-A treatment of CD in adults. These studies included a phase 3, double-blind, randomized, active-control (onaBoNT-A), non-inferiority study conducted in the European Union [19]; a phase 3, randomized, double-blind, placebo-controlled study of fixed doses in the USA (NCT00407030) [20,21]; a phase 4 study in Germany with flexible injection intervals and dosing (NCT00541905) [27]; and a phase 4 randomized, non-inferiority study in the USA comparing two injection schedules (shorter interval of 8 ± 2 weeks vs. longer interval of 14 ± 2 weeks) (NCT01486264) [28]. The main details of the four studies included in the analysis are summarized in Table 2. Two studies were randomized, double-blind, multicenter, phase 3 studies and two studies were open-label phase 4 studies. One study was placebo-controlled [20] and one study had an active control group [19]. Two studies enrolled patients who had prior treatment with botulinum toxin [19,26], while the other two studies included both previously treated and BoNT-A naïve patients. All pre-treated subjects had their last BoNT injection at least 10 weeks prior to study entry and their first incoBoNT-A injection. The number of injection cycles of incoBoNT-A varied from 1 [19] to 11 [28]. Two studies had a main period after a single injection of up to 20 weeks [20] or 24 weeks [27], followed by a long extension period. Both the total dose of incoBoNT-A and the interval between injection sessions were fixed in some studies and flexible in others.

All four studies used the TWSTRS [48,49]. The TWSTRS provides a total score (range 0–85) and three subscores for CD severity (range 0 = mild to 35 = severe), disability due to CD (range 0–30), and CD-related pain (range 0–20). The TWSTRS pain score is based on the patient’s subjective assessment and has three items: severity of pain, duration of pain, and degree of disability due to pain. In the pain severity score, the usual, worst, and least levels of pain during the last week are each rated on an 11-point scale (range 0–10). The pain severity score is calculated as (2 x usual + best + worst)/4 and has a maximum score of 10 points. Duration of pain and disability due to pain are each scored on 6-point scales (range 0–5). The total pain score is calculated as the sum of the three scores, with lower values indicating less pain.

The TWSTRS severity subscale was rated by patients in three studies [20,27,28] and used in the present analysis. Patients in the study by Benecke et al. [19] only completed the TWSTRS disability subscale, but they used a 100 mm pain VAS ranging from 0 (no pain) to 100 (worst possible pain) to rate their present sensation of pain.

### 5.2. Analyses

This pooled analysis evaluated data only for incoBoNT-A-treated patients with pain at baseline. We did not compare incoBoNT-A results with placebo or active control as these data were derived from one study each. Data for different incoBoNT-A dosages used across the studies were pooled, as the change in TWSTRS pain scores did not differ between groups in the studies using different doses or dosing intervals of incoBoNT-A [20,28].

Data were analyzed at the IV and at the CV 4 weeks (±3 or 7 days) after each injection of incoBoNT-A.

This pooled analysis focused on pain severity data, including TWSTRS severity scores (range 0–10) and pain VAS scores (range 0–100), which were transformed to a scale of 0–10 by dividing by 10. Pain severity was categorized as no pain (score = 0), mild pain (score >0 to <3.5), moderate pain (score 3.5 to <6.5), or severe pain (score 6.5–10). The proportion of responders was calculated with response defined as ≥30% or ≥50% reduction from baseline in the pain severity score, reflecting at least moderate or substantial clinically important improvements, respectively [32]. The percentage of patients with complete pain relief (pain score = 0) was also evaluated at each IV and CV.

Data are presented using descriptive statistics as mean (SD) or *n* (%). Change in pain severity from baseline to CV1 (Week 4 in the first injection cycle) was assessed using one-sample *t*-tests.

Since concomitant pain relief medications (analgesics and non-steroidal anti-inflammatory drugs) were allowed in all four studies, with no restrictions on dosing and frequency of intake, the percentage of patients using concomitant pain medication at each IV and CV was determined. As a sensitivity analysis, we examined pain severity responses in the subgroup of patients not taking concomitant pain-relieving medication.

All analyses were performed using Statistical Analysis Software (SAS) version 9.4.

## Figures and Tables

**Table 1:** Baseline characteristics of patients with pain at baseline.

Characteristic	Total (*n* = 678)	Pain Severity at Baseline ^a^		
		Mild (*n* = 247)	Moderate (*n* = 291)	Severe (*n* = 140)
Age (years)	53.6 ± 11.5	53.4 ± 11.8	53.7 ± 11.6	54.0 ± 10.8
Female	463 (68.3)	152 (61.5)	203 (69.8)	108 (77.1)
Ethnicity				
White	640 (94.4)	242 (98.0)	271 (93.1)	127 (90.7)
Black or African American	16 (2.4)	2 (0.8)	8 (2.8)	6 (4.3)
Asian	5 (0.7)	0	3 (1.0)	2 (1.4)
Other/missing	17 (2.5)	3 (1.2)	9 (3.1)	5 (3.5)
Height (cm)	167.7 ± 9.0	168.2 ± 9.2	167.7 ± 8.9	166.5 ± 9.0
Weight (kg)	75.1 ± 16.2	75.3 ± 15.9	75.1 ± 15.9	74.9 ± 17.8
Disease severity ^b^				
Mild	168 (24.8)	81 (32.8)	70 (24.1)	17 (12.1)
Moderate	365 (53.8)	131 (53.0)	162 (55.7)	72 (51.4)
Severe	145 (21.4)	35 (14.2)	59 (20.3)	51 (36.4)
BoNT naïve	123 (18.1)	29 (11.7)	57 (19.6)	37 (26.4)
Years since CD diagnosis	9.2 ± 7.9	9.2 ± 7.4	9.3 ± 8.0	9.0 ± 8.6
Pain severity score	4.26 ± 2.32	1.73 ± 1.06	4.91 ± 0.80	7.40 ± 0.83

Data presented as mean ± standard deviation or *n* (%). ^a^ Pain severity score (range 0–10): mild = >0 to <3.5; moderate = 3.5 to <6.5; severe = 6.5–10. ^b^ Calculated from TWSTRS severity score: mild = 0–15, moderate = 16–21, severe = 22–35. BoNT, botulinum toxin; CD, cervical dystonia; TWSTRS, Toronto Western Spasmodic Torticollis Rating Scale.

**Table 2:** Details of the four studies included in the pooled analysis.

Study Name, NCT Number and Reference(s)	Phase	Study Design & Objectives	Patients & Indication	Treatments	Primary Efficacy Outcome Measure	Pain Measures
Benecke et al., 2005 [19]	3	Randomized, double-blind, active-controlled, parallel-group, multicenter, non-inferiority study in the EU to investigate the safety and efficacy of incoBoNT-A compared to onaBoNT-A in patients with CD.	*n* = 463 Adults with CD (predominantly of the rotational form; i.e., spasmodic torticollis) who had shown stable responses in at least two previous onaBoNT-A injection sessions prior to study entry. Last injection session at least 10 weeks prior to randomization. Baseline TWSTRS severity score ≥10.	One i.m. injection session of incoBoNT-A (*n* = 231) or onaBoNT-A (*n* = 232), 70–300 U at baseline visit (dose equivalent to last two injection sessions).	Change in CD severity using the TWSTRS severity score from baseline to control visit on day 28 ± 7 after injection of study medication. Total study duration: 16 weeks.	TWSTRS disability subscale score (range 0–5); pain VAS score (0–100 mm)
NCT00407030 Comella et al., 2011 [20] Evidente et al., 2013 [21]	3	Prospective, double-blind, placebo-controlled, randomized, multicenter study in the USA with a double-blind parallel-group extension period to investigate the efficacy and safety of different doses of incoBoNT-A in the treatment of CD.	*n* = 233 (main period); *n* = 217 (extension period) Adults with CD of predominantly rotational form (i.e., spasmodic torticollis). At least 40% BoNT-A-treatment naïve Pre-treated subjects had to be stable and have their last BoNT injection at least 10 weeks prior to study entry. Baseline TWSTRS total score ≥20, TWSTRS severity score ≥10, TWSTRS disability score ≥3, TWSTRS pain score ≥1.	Main period: One injection session of IncoBoNT-A (120 U or 240 U; fixed total dose) or placebo. Extension period: Up to 5 additional injection cycles of IncoBoNT-A (120 U or 240 U; fixed total dose). Interval between injection sessions: 6–20 weeks.	Change in TWSTRS total score from baseline to Week 4 (±3 days) after injection. Total study duration: 88 weeks.	TWSTRS pain score (range 0–20)
NCT00541905 Dressler et al., 2013 [27]	4	Prospective, open-label, single-arm, multicenter study in Germany to investigate the long-term efficacy and safety of incoBoNT-A in patients with CD.	*n* = 76 Adults with CD of predominantly rotational form (i.e., spasmodic torticollis). Baseline TWSTRS total score ≥25, TWSTRS severity score ≥10 and TWSTRS disability score ≥3 25% naïve to BoNT-A. Pre-treated patients had shown stable response and the most recent treatment was ≥10 weeks prior to first injection.	Main period: One injection session Flexible dosing: ≤300 U total dose; ≤50 U per injection site. Extension period: Four additional injection sessions. Interval between injection sessions: 10–24 weeks.	Change in TWSTRS total score from baseline to Week 4 after 1st injection. Total study duration: 51–121 weeks.	TWSTRS pain score (range 0–20)
NCT01486264 Comella et al., 2022 [28]	4	Prospective, open-label, randomized, multicenter, non-inferiority study in the USA (CD-FLEX) evaluating two dosing schedules of incoBoNT-A in patients with CD.	*n* = 282 Adults with CD who reported therapeutic benefit from previous BoNT treatment. All pre-treated with at least two successful BoNT injections; most recent treatment at least 12 weeks before enrolment.	Short Flex: injection interval 8 ± 2 weeks (*n* = 142) Long Flex: injection interval 14 ± 2 weeks (*n* = 140). Initial dose comparable to most recent BoNT dose (±10%) and to remain stable thereafter. Up to 11 injection cycles 10 visits.	Change in CD severity using the TWSTRS severity score from baseline to 4 weeks after 8th injection Overall mean duration was 452.4 days for the Short Flex group and 691.0 days for the Long Flex group. Mean duration of cycles was 55.1 days for the Short Flex group and 86.4 days for the Long Flex group (full analysis set).	TWSTRS pain score (range 0–20)

BoNT, botulinum toxin; CD, cervical dystonia; EU, European Union; incoBoNT-A, incobotulinumtoxinA; i.m, intramuscular; onaBoNT-A, onabotulinumtoxinA; TWSTRS, Toronto Western Spasmodic Torticollis Rating Scale; VAS, visual analog scale.

## Data Availability

Data are not publicly available.

## Supplementary Materials

The following supporting information can be downloaded at: https://www.mdpi.com/article/10.3390/toxins15050333/s1, Figure S1: Number of incobotulinumtoxinA-treated patients with pain severity assessed during injection cycles 1–5 for the total population (numbers given outside each bar) and by study; Figure S2: Percentage of patients in each pain severity category at baseline and control visit 1 (CV1) for: (a) total population (*n* = 669) and (b) subgroup of patients not taking concomitant pain medication (*n* = 379); Figure S3: Category of pain severity at each visit following multiple injections of incobotulinumtoxinA. The percentages in each bar are based on the number of patients with a pain severity assessment at that visit (*n* value). CV, control visit IV, injection visit; Table S1: Number of incobotulinumtoxinA-treated patients with pain severity assessed at the injection visit (IV) and control visit (CV) of each injection cycle for the total population and by individual study; Table S2: Use of concomitant pain medication in the first injection cycle by patients who had pain at baseline and data available on pain medication use.
