# Supplementary material for: Pain Reduction in Cervical Dystonia Following Treatment with IncobotulinumtoxinA: A Pooled Analysis
Source: Toxins (Basel). 2023 May 12;15(5):333. doi: 10.3390/toxins15050333 (PMC10224129; doi:10.3390/toxins15050333)

Supplementary Material

Supplementary Table S1 Number of incobotulinumtoxinA-treated patients with pain severity assessed at the injection visit (IV) and control visit (CV) of each injection cycle for the total population and by individual study.

Study visit	Total	Benecke et al. 2005 [19]	Comella et al. 2011 [20]	Dressler et al. 2013 [27]	Comella et al. 2022 [28]
IV1	678	191	226	72	189
CV1	669	189	222	71	187
IV2	465	0	208	68	189
CV2	263	0	197	66	0
IV3	435	0	181	65	189
CV3	235	0	172	63	0
IV4	411	0	160	63	188
CV4	215	0	153	62	0
IV5	371	0	122	61	188
CV5	179	0	112	57	10
IV6	248	0	61	0	187
CV6	59	0	59	0	0
IV7	188	0	0	0	188
CV7	16	0	0	0	16
IV8	189	0	0	0	189
CV8	189	0	0	0	189
IV9	10	0	0	0	10
CV9	0	0	0	0	0
IV10	5	0	0	0	5
CV10	0	0	0	0	0
IV11	4	0	0	0	4
CV11	0	0	0	0	0

Supplementary Table S2 Use of concomitant pain medication in the first injection cycle by patients who had pain at baseline and data available on pain medication use. IV, injection visit; CV, control visit.

	**Total *n***	***n* (%) taking concomitant pain medication**
IV1	678	243 (35.8)
Mild pain	247	62 (25.1)
Moderate pain	291	118 (40.6)
Severe pain	140	63 (45.0)
CV1	669	250 (37.4)
No pain	69	15 (21.7)
Mild pain	321	97 (30.2)
Moderate pain	223	109 (48.9)
Severe pain	56	29 (51.8)
Patients with ≥30% pain reduction at CV1	322	94 (29.2)
Patients with ≥50% pain reduction at CV1	230	61 (26.5)

Supplementary Figure S1 Number of incobotulinumtoxinA-treated patients with pain severity assessed during injection cycles 1–5 for the total population (numbers given outside each bar) and by study. IV, injection visit; CV, control visit.

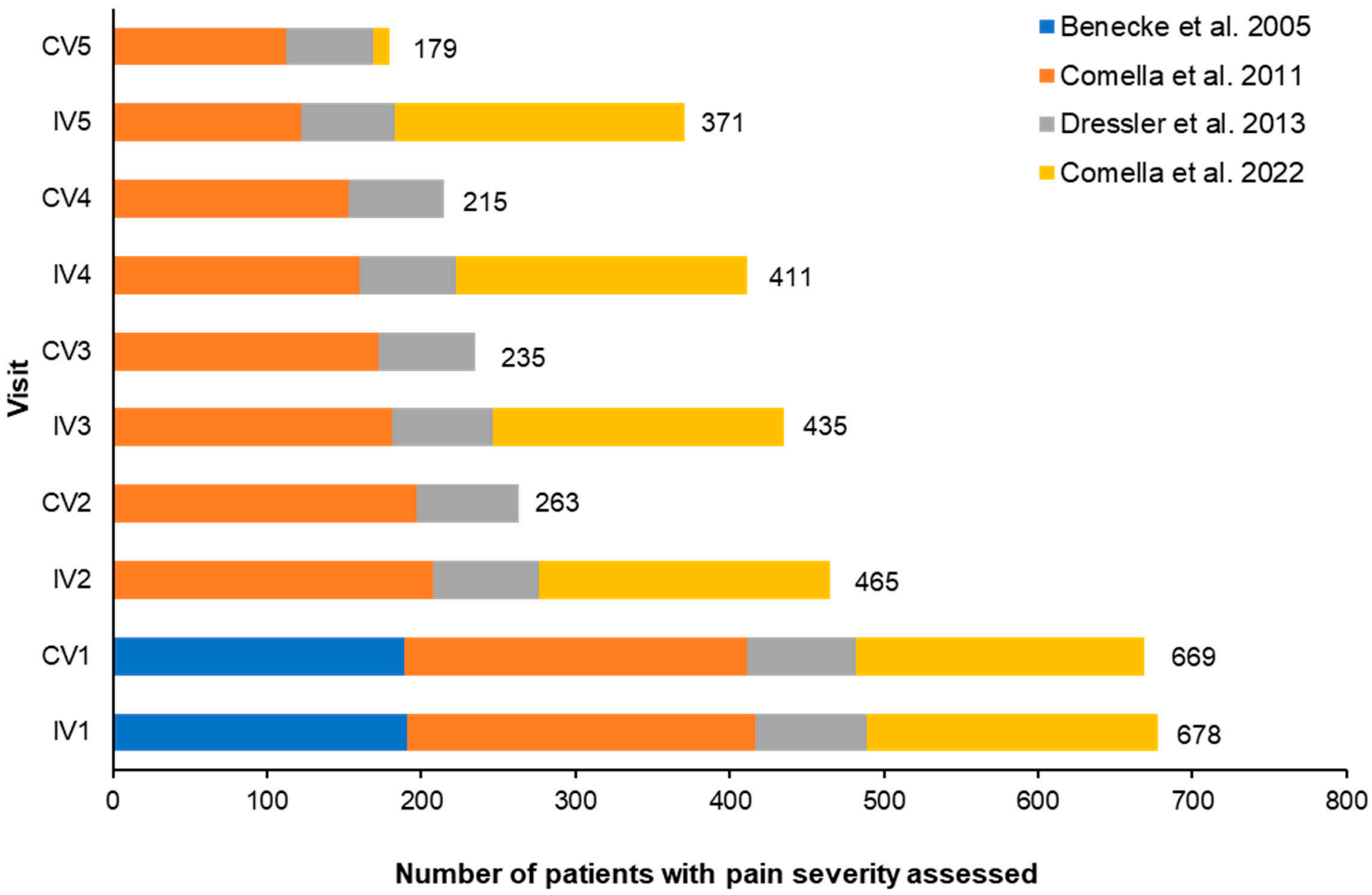

Supplementary Figure S2 Percentage of patients in each pain severity category at baseline and control visit 1 (CV1) for (A) total population (N=669) and (B) subgroup of patients not taking concomitant pain medication (*n*=379)

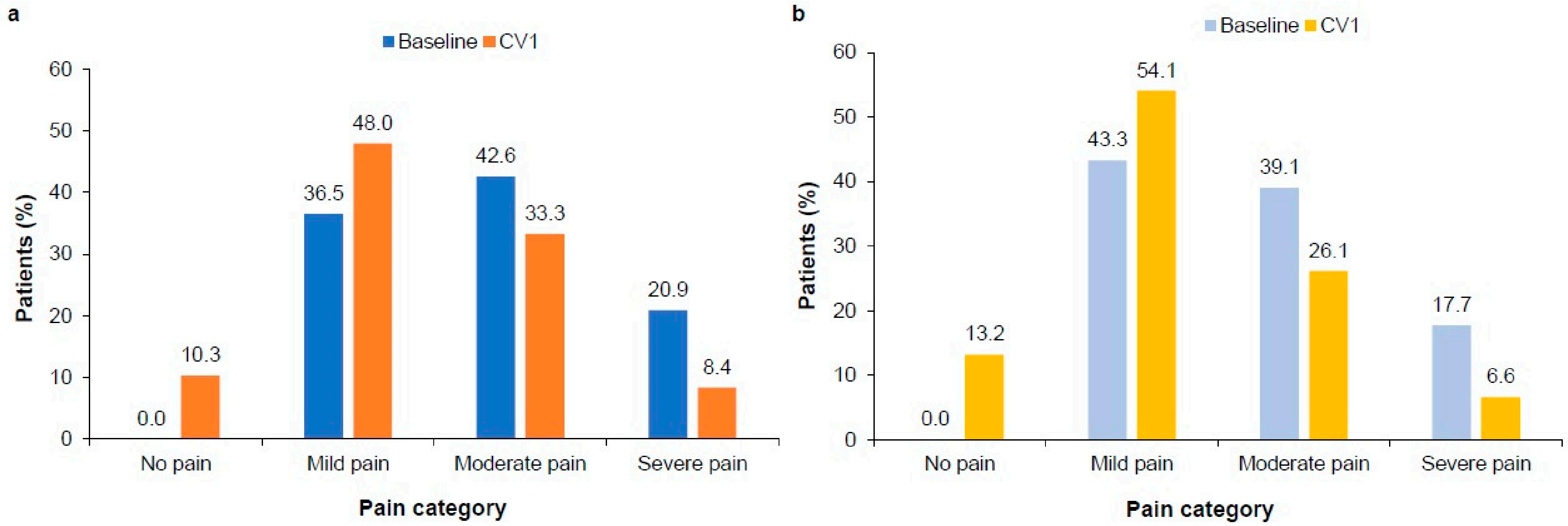

Supplementary Figure S3 Category of pain severity at each visit following multiple injections of incobotulinumtoxinA. The percentages in each bar are based on the number of patients with a pain severity assessment at that visit (*n* value). CV, control visit, IV, injection visit.

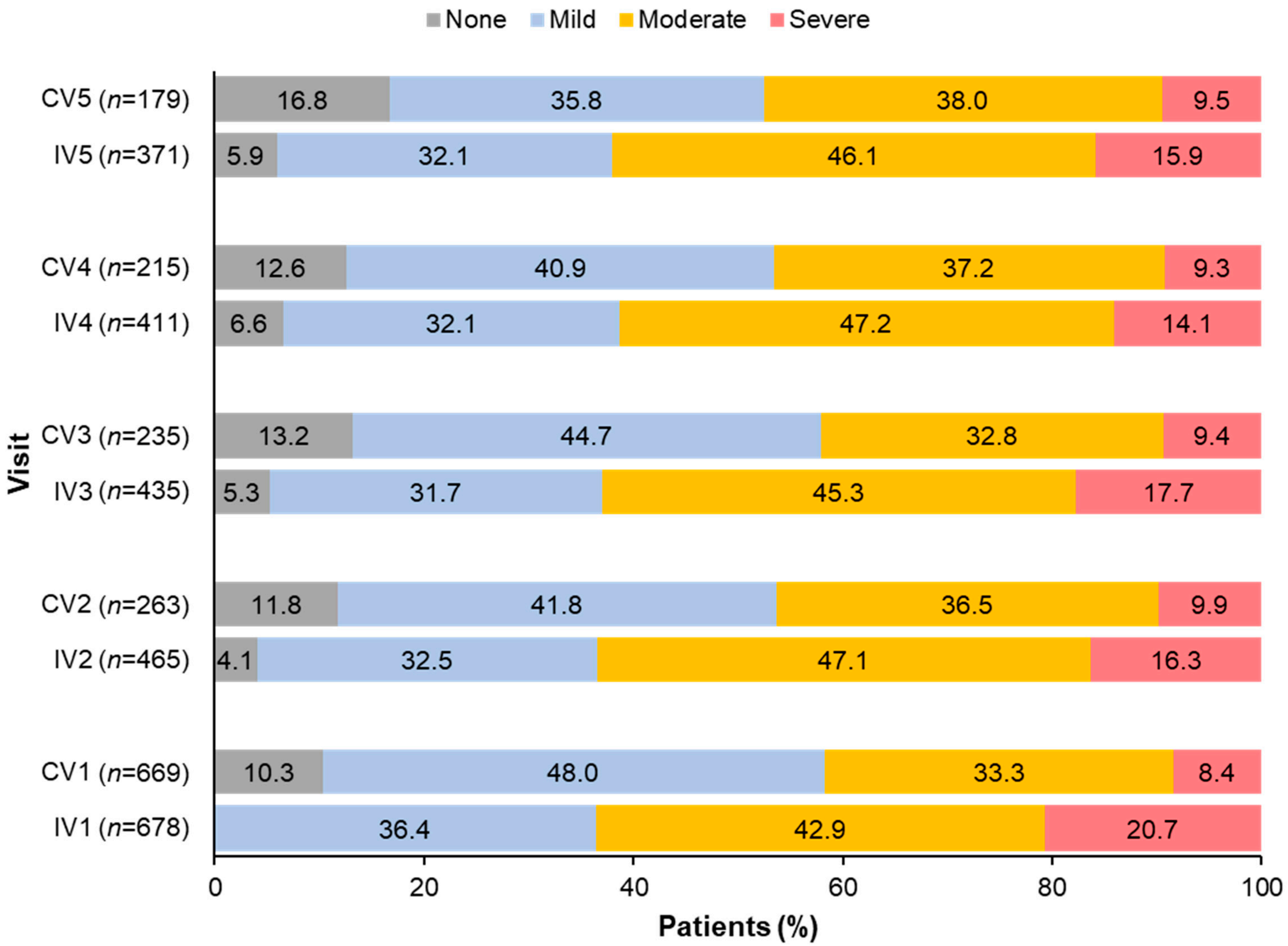

Supplement: Supplementary file 1 [file toxins-15-00333-s001.zip › toxins-2360153-supplementary.pdf]
